# Quality Control of High Carbon Steel for Steel Wires

**DOI:** 10.3390/ma12060846

**Published:** 2019-03-13

**Authors:** Wei Yan, Weiqing Chen, Jing Li

**Affiliations:** State Key Laboratory of Advanced Metallurgy, University of Science and Technology Beijing, Beijing 100083, China; chenweiqing@metal.ustb.edu.cn (W.C.); lijing@ustb.edu.cn (J.L.)

**Keywords:** high-carbon steel, segregation, inclusion, solidification structure

## Abstract

High-carbon steel wires used for bridge cables, tire reinforcement materials and cutting materials of silicon ingot for photovoltaic industry require an extremely fine diameter and high strength. Poor control of centerline segregation, inclusion and microstructure of high-carbon steel is detrimental to drawability and subsequent fatigue performance. Prof. Weiqing Chen’s group at the University of Science and Technology Beijing (USTB) has been investigating the quality control of high-carbon steel through a low-cost one-stage hot rolling process since 2000. This paper reviews the main research from this group. The laboratory-scale and industrial results are presented. Intensive secondary cooling, final electromagnetic stirring (F-EMS), final permanent magnetic stirring (F-PMS), and soft reduction are investigated and applied to control centerline segregation, and the application scope is also discussed. A combination of redesign of submerged entry nozzle (SEN) and refining slag, utilization of Al-free refractory and the addition of low-melting-point compounds is studied and applied effectively to control inclusions. Measurements and mechanisms to control network cementite, martensite, banded structure and undesired texture are investigated and discussed. Integration of the above research has achieved industrial application in more than 10 steelworks and was further extended to application in spring steel, welding wire steel and some other wire rods.

## 1. Introduction

High-carbon steel wires produced through hot rolling and cold drawing of steel billets are utilized extensively for pre-stressed concrete, bridge cables, wire ropes, etc. They are also used to produce steel cords for tire reinforcement in motor vehicles, and in saw wires for cutting silicon panels for the photovoltaic industry. For instance, high-carbon steel wire rods for saw wires have to be drawn into steel wires with a diameter as low as 0.08 to 5.5 mm [1], and there must be no breakage within 250 km during drawing along with extremely high strength to meet the rigorous utilization demands [2]. The finer diameter, lower breakage rate, and higher strength require better quality for master steel billets. It is generally accepted that the quality of the final products largely depends on the quality of wire rods and steel billets, including centerline segregation, cleanness, microstructure, etc., due to the heredity of these quality defects.

Centerline segregation, especially for high-carbon steel, is one of the key quality defects, which leads to non-uniformity in inner compositions and mechanical properties, and has a negative influence on the drawability and fatigue performance of final products. Redistribution of solute elements like carbon at the solid-liquid interface during solidification is the main reason for centerline segregation [3,4]. The steel grades used to produce steel wire strands, steel cords and saw wires have a high carbon content of ~0.8 wt.% or even 0.9 wt.%. The high carbon content is the first factor to aggravate centerline segregation. In the meantime, the centerline carbon segregation is always accompanied by a decreasing ratio of fine pearlitic microstructure, increasing rates of brittle and continuous network cementite, hard martensite, central porosity, and shrinkage, which will further decrease the compactness and mechanical properties of products, and, eventually, increase breakage frequency during drawing. The point is to highlight that the centerline carbon segregation is barely able to be eliminated during heat treatment or rolling [5,6]. As a consequence, controlling the centerline carbon segregation of high-carbon steel before rolling is a fundamental strategy to improve the quality of the final products.

Low cleanness is another key quality issue that not only results in an increase in the breakage rate, but also exacerbates the fatigue characteristics. On the one hand, impurity elements like [O], [N], [P], [S], etc. are required to be removed to extremely low levels for high-carbon steel through the combination of blowing and refining. On the other hand, the mechanical properties of high-carbon steel are closely related to the size, amount, and deformability of inclusions because some large and undeformable inclusions (inclusions with a length/width ratio larger than three are defined as deformable inclusions according to an international standard ISO 4967 and a China standard GB/T-10561, the length and width can be determined through measuring the scanning electron microscope (SEM) image of inclusion using Image Pro Plus (IPP) software) always initiate cracking and induce the breakage of steel wires during drawing [7,8]. Therefore, inclusions should be controlled to be few, fine and deformable. Compared with the removal of impurity elements, control of inclusion to a strict level is more difficult and important. In practice, control of inclusions runs through the whole process from steelmaking to rolling. Consequently, many measurements, including: (a) limiting the generation of inclusions through controlling [O], [N], [Al], [Ti], etc. and avoiding secondary oxidation [9,10,11], (b) limiting the entrapment of inclusions through suppressing slag carry-over and slag entrapment as well as refractory erosion [8,10,11], (c) promoting the removal of inclusions through optimizing the flow field and accelerating floatation and absorption [12,13,14], (d) improving the deformability of inclusions through using a suitable deoxidation process and redesigning the refining slag [9,15,16,17], have been investigated to control the inclusions during the production of high-carbon steel wire rods.

In addition to central segregation and inclusions, structure control of wire rods is also crucial for improvement of product quality because the structural defects of wire rods after hot-rolling from steel billets can be inherited into the steel wires and appear as quality issues. Network cementite, martensite, pearlite, band structure, texture, etc., are important structures of high-carbon steel wire rods [18,19,20]. Generally, high-carbon steel for the production of steel wire strands, steel cords and saw wires is hypereutectoid steel with a carbon content of more than 0.78 wt.%; a hypereutectoid transformation will happen during cooling and secondary cementite will precipitate along the grain boundary to form a continuous or discontinuous network. Fundamentally, secondary cementite stems centerline carbon segregation; it is so brittle that it can separate grains and reduce the binding force among the grain boundary [21]. It further decreases the strength and ductility of wire rods. Martensite can also be precipitated, depending on the cooling scheme. Martensite is a structure with high strength and hardness, and the hardness increases with the increase in carbon content. The strong and hard martensite will induce stress and further cause fractures along martensite during drawing from wire rods to wires [18]. Precipitation of fine pearlite will effectively alleviate this issue. A banded structure is a structure with a different property from the matrix, which may also induce stress and deteriorate the quality [22,23]. Texture is a structure that represents the preferred orientation of grains during deformation. Intensive drawing is necessary to produce steel wires from wire rods. If the preferred orientation deviates from the optimal slippage direction of grains, the drawing will be obstructed [20,24]. Therefore, control of structure of wire rods is important to achieve better product quality.

It is apparent that the above issues are crucial for the successful production of high-carbon steel products. However, before 2000, only a few steelworks in China could produce high-carbon steel like 82B (SWRH82B, 0.79–0.86 wt.% C, 0.15–0.35 wt.% Si, 0.3–0.6 wt.% Mn, ≤0.03 wt.% P, ≤0.03 wt.% S) for steel strands through bloom casting and subsequent two-stage hot rolling (continuous casting–hot rolling–hot rolling–high-speed wire rolling); almost no steelworks can produce tire cord steel and saw wire steel. Based on the situation at that time, Prof. Weiqing Chen’s group from the University of Science and Technology Beijing (USTB) conducted systematic research on a low-cost one-stage hot rolling process of billet, including steelmaking, refining, casting, single hot rolling, and cold drawing, to produce and control the quality of high-carbon steel for steel strands, steel cords, and saw wires since 2000. A series of methods was developed and they have been applied in more than 10 steelworks to produce high-carbon steel for steel strands, steel cords and saw wires. In addition, they were also applied in the production of other wire rods like spring steel, welding wire steel, cold heading steel, etc. In this paper, the crucial control techniques of centerline segregation, inclusion and structure of high-carbon steel that were investigated and developed by Prof. Chen’s group are summarized.

## 2. Control of Centerline Segregation

Increasing the carbon content in steel can improve the strength, but also causes severe problems like centerline segregation. The centerline segregation, especially centerline carbon segregation of high-carbon steel, caused by solute redistribution during solidification results in breakage during the drawing or twisting of steel wires. The theory below has been proposed to explain the segregation mechanism. Columnar fronts always advance to the center and form bridges, which prevents fresh liquid from feeding the shrinkage cavities. This leads to suction of impure interdendritic liquid from the surrounding mushy zone into the central region, thus increasing centerline segregation [3,4]. Suppressing solute segregation, enlarging the equiaxed zone and enhancing liquid feeding are beneficial to reduce central segregation.

### 2.1. Control of Centerline Segregation Using Intensive Secondary Cooling

**Figure 1:**
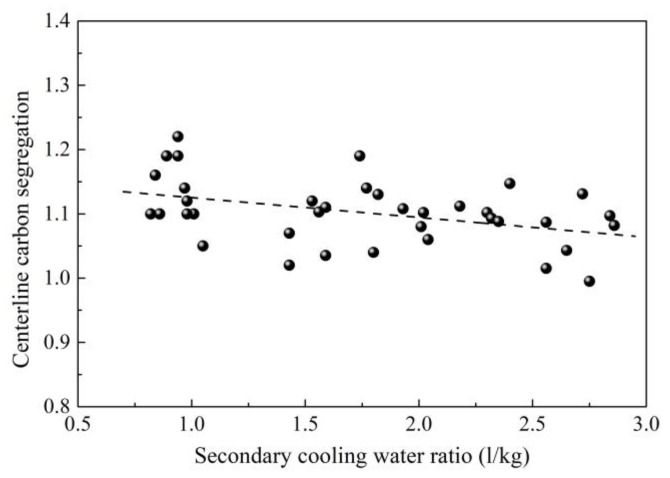
Effect of secondary cooling water ratio on centerline carbon segregation. Adapted from [29].

Increasing the cooling intensity may be helpful because a high cooling rate can prevent solute precipitation and refine grains. According to this theory, intensive cooling was tried first in continuous casting of 82B billet [25,26]. In order to determine centerline carbon segregation, the following method was adopted. A total of 20 steel pieces with a thickness of 25 mm were cut from a casting billet. Then, the steel pieces were etched using hot aqueous hydrochloric acid solution with a volume ratio of 1:1. After this, the metal filings were obtained through drilling holes using a drill with a diameter of 5 mm at the geometric center of steel pieces. Then, the metal filings were analyzed to determine the C content. Finally, the centerline carbon segregation can be calculated through the mass ratio of average C content of metal fillings to C content of steel in tundish. It can be seen from Figure 1 that the centerline carbon segregation decreases when increasing the secondary cooling water ratio [27,28,29]. However, the correlation is not strong enough. Although increasing secondary cooling water ratio to decrease centerline carbon segregation was verified to be effective during industrial production, this does not mean that centerline carbon segregation of all billets under low secondary cooling water ratio is high, similarly, not all billets under high secondary cooling water ratio has low centerline carbon segregation during industrial production due to the uncertainties in the complex production process. This may decrease the correlation between secondary cooling water ratio and segregation. In addition to this, although an evaluation standard of centerline segregation was set to evaluate the centerline segregation, few researchers evaluated its accuracy. Moreover, if the billet is not strictly square due to uneven cooling, it will be difficult to determine the center of billet, this also causes error. More data are also beneficial for increasing the accuracy.

It is well known that a single increase of secondary cooling water ratio cannot totally suppress segregation. There should be an application range in section size. However, the application range of intensive secondary cooling is still unknown. Further study was conducted by Chen’s group to probe the effective range for a billet to suppress centerline segregation through single intensive secondary cooling [29].

**Figure 2:**
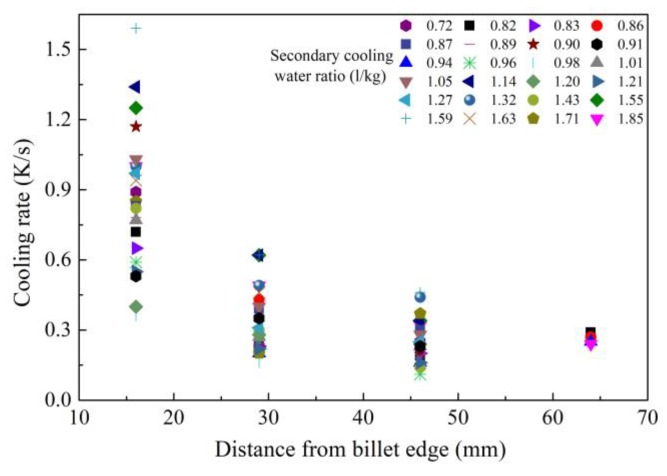
Relationship between cooling rate and distance from billet edge. Adapted from [29].

The cooling rates at various positions of billets were calculated based on the relationship with secondary dendritic arm spacing (SDAS) to study the change with distance from billet edge. The SDAS were measured using IPP software based SEM images of solidification structure taken from the corresponding position. As shown in Figure 2 [29], it shows the relationship between distance from billet (with a section of 150 × 150 mm) edge and cooling rate at the position. The symbols represent different secondary cooling water ratios with a range of 0.72–1.85 L/kg. It is clear that the cooling rate decreases and tends to be consistent with increasing distance from the billet edge. The differences of cooling rate at 16, 29 and 46 mm from the billet edge are 1.23, 0.46 and 0.15 K/s, but the difference decreases to 0.05 K/s at a distance of 64 mm from the billet edge. This indicates that increasing the secondary cooling water ratio cannot change the cooling rate once and for all, so single intensive secondary cooling is no longer effective to suppress centerline carbon segregation for a billet with a section size larger than 130 × 130 mm. 

**Figure 3:**
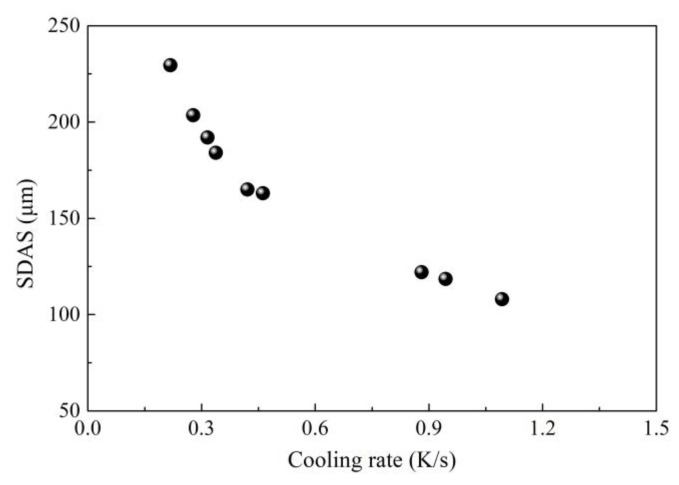
Relationship between secondary dendritic arm spacing (SDAS) and cooling rate. Adapted from [29].

**Figure 4:**
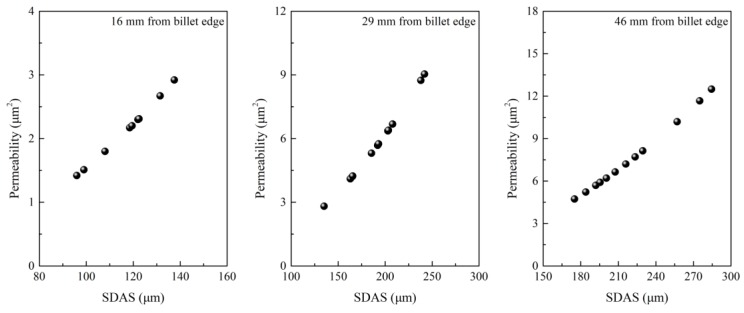
Relationship between permeability and secondary dendritic arm spacing. Adapted from [29,30].

**Figure 5:**
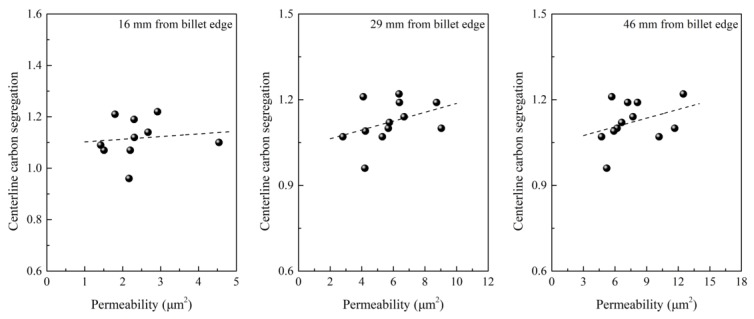
Relationship between centerline carbon segregation and permeability. Adapted from [29,30].

The effect of intensive cooling on centerline carbon segregation was also investigated through constructing a relationship between the cooling rate, SDAS, the permeability of the mushy zone, and centerline carbon segregation. As shown in Figure 3, Figure 4 and Figure 5, an increase in the cooling rate shortens SDAS, which reduces the permeability of the mushy zone and then in turn reduces the centerline carbon segregation [29,30]. Generally, the permeability of the mushy zone is of importance for the transport of liquid phase and solute; a reduction in permeability will suppress the transport of the liquid phase and solute, and SDAS is a main micro-parameter that affects permeability [30,31,32]. In spite of this, it is noted that the correlation between centerline carbon segregation and permeability is also not strong enough according to industrial results. More factors can decrease the accuracy because the permeability was calculated according to the Kozeny–Carman equation. Some parameters used during calculation of permeability like the Kozeny–Carman constant, interface concentration, interface area and solid fraction were estimated according to references, which may decrease the correlation. 

### 2.2. Control of Centerline Segregation Using F-EMS

As concluded above, intensive secondary cooling can only play a slight role in controlling centerline segregation of a high-carbon steel billet with a section size of larger than 130 × 130 mm, especially for a section size larger than 140 × 140 mm. However, most steel plants hope to produce high-carbon steel billets with larger sections of 150 × 150 mm or 160 × 160 mm in order to increase productivity. Suppressing bridging among columnar grains during solidification and enrichment of solute are main measurements to control centerline segregation. Final electromagnetic stirring (F-EMS) can break the dendrites into fragments, which act as a further nucleation site to enlarge the equiaxed zone, and meanwhile suppress the flow of liquid phase into the center, which is beneficial for the suppression of segregation. A series of studies have been conducted to explore the effect of F-EMS on the segregation of high-carbon steel in our group [25,26,27,28,29,33,34,35,36,37,38].

**Figure 6:**
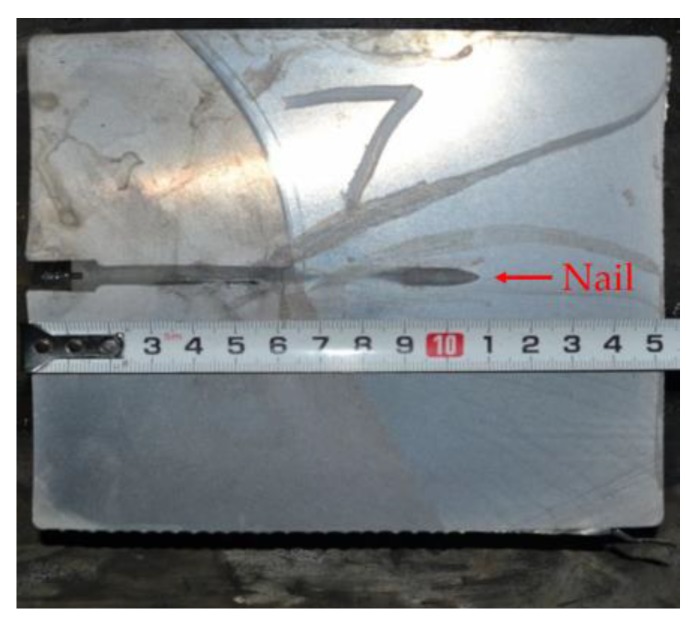
Section morphology of billet after nail shooting. The picture was taken using a digital camera. Adapted from [38].

**Figure 7:**
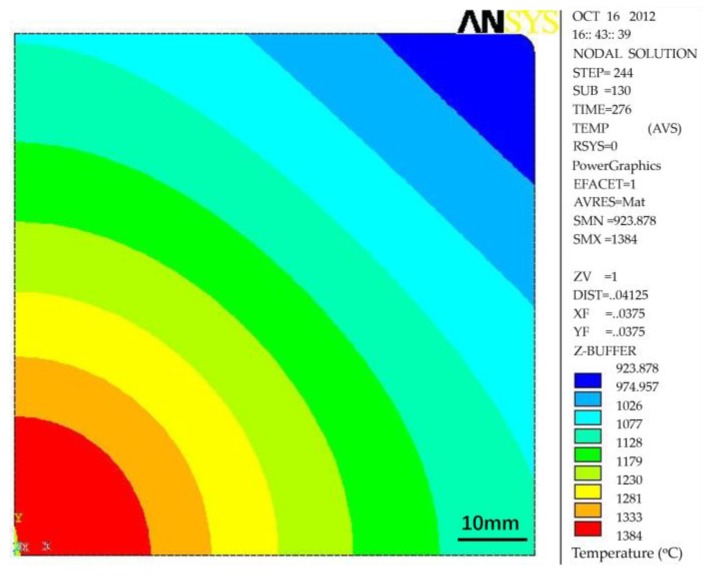
Distribution of temperature field of billet at position of nail shooting. Adapted from [38].

First, the installation position of final electromagnetic stirrer is crucial for achieving the best stirring effectiveness. As the stirrer comes into play through stirring the mushy zone, the liquid core size or solidification ratio (1 − (liquid core size/billet thickness)) of billet is always used to reflect the installation position. Whether the installation position is suitable or not can be evaluated through the improvement effectiveness of the billet quality. Generally, mathematical modeling or nail shooting are employed to determine the liquid core size or solidification ratio [25,26,29,33]. After nail shooting, the billet was cut and machined gradually to reveal the nail. Figure 6 presents the nail shooting result of an 82B high-carbon steel billet with a section of 150 × 150 mm [39]. The corresponding temperature field distribution at the same position obtained through mathematical modeling is also presented in Figure 7 [38]. The thicknesses of the liquid core and the solidified shell determined through nail shooting are 26 and 62 mm, respectively, which are nearly equal to 24 and 63 mm, the thicknesses of the liquid core and the solidified shell determined through mathematical modeling. Generally, a solidified ratio of 0.7–0.8 is recommended for installation of the final electromagnetic stirrer according to the evaluation of billet quality [25,26]. The installation position can therefore be determined conveniently through mathematical modeling.

**Figure 8:**
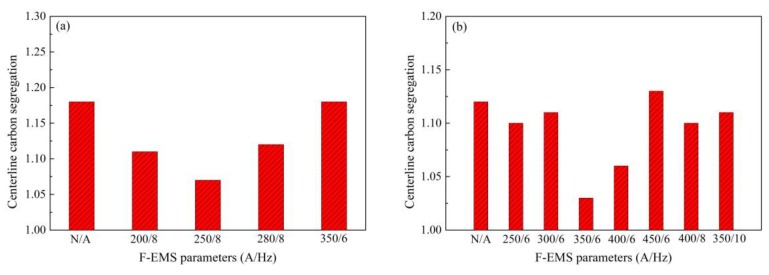
Effect of stirring parameters on centerline carbon segregation of 82B steel. (**a**) 150 × 150 mm, (**b**) 160 × 160 mm. Adapted from [29].

Stirring current and frequency are key parameters for F-EMS. Figure 8 shows the effects of stirring current and frequency on the centerline carbon segregation of 82B high-carbon steel with different sections [29]. Apparently, both the high/low stirring current and no F-EMS will cause high centerline carbon segregation; the best stirring current and frequency is 250 A/8 Hz for 82B steel with a section of 150 × 150 mm (Figure 8a). However, the best stirring current and frequency are 350 A/6 Hz for 82B steel with a section of 160 × 160 mm (Figure 8b). This indicates that F-EMS can control centerline carbon segregation well, but the stirring parameters should be adjusted for different section sizes. Compared with single intensive secondary cooling, combined application of F-EMS and intensive secondary cooling can control centerline segregation of high-carbon steel billet with a larger section size. This is because the final electromagnetic stirrer is always installed at a position where the liquid core size range of billet is about 30 to 50 mm, this liquid core size exactly compensates for the shortage of single intensive secondary cooling in the section range (≤130 × 130 mm).

**Figure 9:**
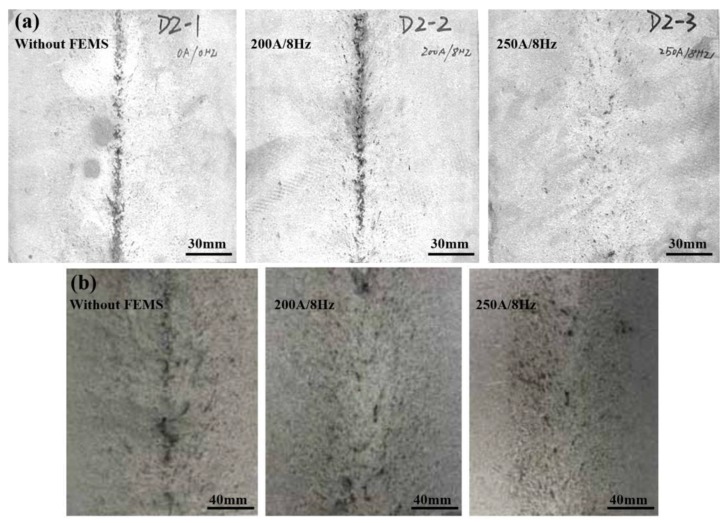
Effect of final electromagnetic stirring (F-EMS) on V segregation. (**a**) 150 × 150 mm, (**b**) 160 × 160 mm. The pictures were taken using a digital camera after etching the steel. Adapted from [29].

In addition to centerline segregation, V segregation is also a typical quality defect of high-carbon steel, which is supposed to cause segregation of cementite along the grain boundary and then results in drawing fracture. Suppression of V segregation is also important for better quality. In order to reveal the macro V segregation, the billet was first cut along casting speed and then machined through a milling machine, followed by etching through hot aqueous hydrochloric acid solution with a volume ratio of 1:1. As shown in Figure 9, F-EMS has a similar effect on V segregation to that on the centerline carbon segregation of high-carbon steel billets with sections of 150 × 150 mm (Figure 9a) and 160 × 160 mm (Figure 9b) [29]. V segregation was improved significantly after application of corresponding stirring parameters, under which the centerline carbon segregation was also improved.

### 2.3. Control of Central Segregation Using F-PMS

F-EMS plays an encouraging role in the control of centerline segregation, but there are some disadvantages that exist with F-EMS, such as high power consumption, necessary intensive cooling for induction coils, huge and complex accessory devices, especially low stirring ability for a large section billet, etc. However, in order to further increase productivity, the billet size was increased from 150 × 150 mm or 160 × 160 mm to 180 × 240 mm. For this billet section, casting and a two-stage hot rolling process are always employed conventionally to ensure product quality. In our study, a casting and one-stage hot rolling process was developed to lower costs and increase productivity. Therefore, more intensive final stirring is necessary for a larger section billet to eliminate centerline segregation. In comparison with electromagnetic stirring, permanent magnetic stirring has stronger stirring intensity, lower energy consumption, simpler structure, lower requirement for cooling, and lower cost. Before the development and application of an industrial permanent magnetic stirrer during the continuous casting of steel in 2014, a permanent magnetic stirrer was always used in non-ferrous metal industries like melting furnace of aluminum [39] or low-melting-point alloy [40,41]. Although Kobayashi [42], Kawami [43], and Hagiwara [44] have conducted industrial pilot tests during steel casting using permanent magnetic stirring, there has been no further industrial application. A permanent magnetic stirrer was developed and applied during our study to control the centerline segregation and internal structure of 82B steel and 82A tire cord steel with a section of 180 × 240 mm [45,46,47,48,49,50]. The method of determining the installation position of permanent magnetic stirrer is the same with the final electromagnetic stirrer.

**Figure 10:**
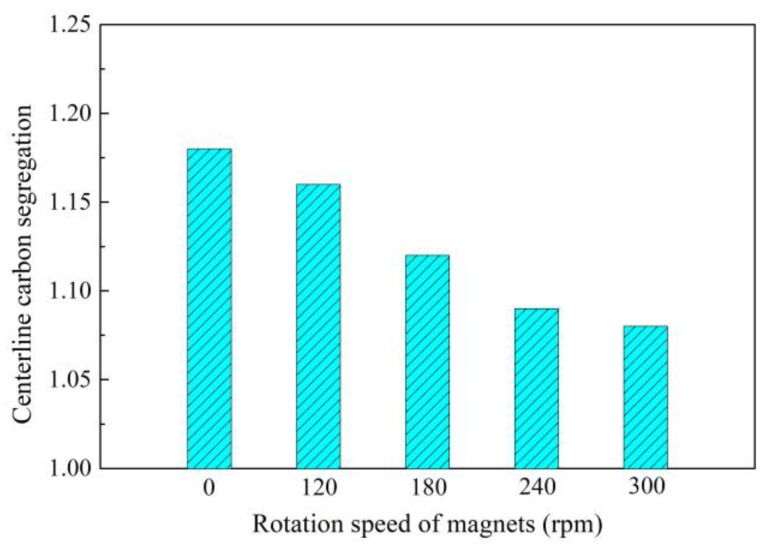
Effect of final permanent magnetic stirring (F-PMS) on centerline carbon segregation of 82A steel billet. Adapted from [45].

Figure 10 [45] shows the change of centerline carbon segregation of 82A steel (with similar composition to 82B but higher requirement for strength and cleanness) with the rotation speed of magnets. It is apparent that the centerline carbon segregation decreases almost linearly from 1.18 to 1.08 with the increase in rotation speed of the magnets from 0 to 300 rpm. The application of final permanent magnetic stirring (F-PMS) remarkably improves the centerline carbon segregation of an 82A rectangular billet. Similar results of low carbon segregation were also obtained from application in continuous casting of 82B steel [46,47].

**Figure 11:**
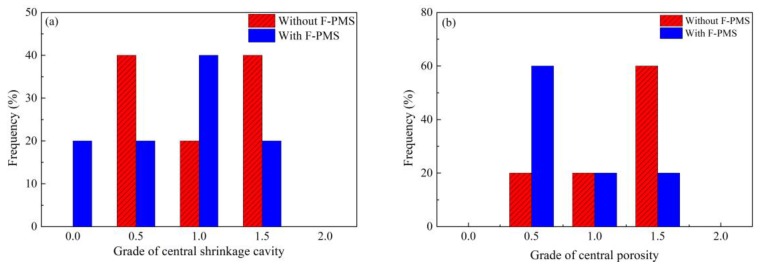
Effect of F-PMS on (**a**) central shrinkage cavity and (**b**) porosity of 82A steel billet. Adapted from [45,51].

Figure 11 [45,51] shows the grade changes of central shrinkage cavity and porosity before and after application of F-PMS. The grades were evaluated according to the following method. The billet was first cut into pieces and then machined through a milling machine, followed by etching through hot aqueous hydrochloric acid solution with a volume ratio of 1:1. Finally, the etched steel pieces were compared with standard pictures from China standard for ferrous metallurgy (Standard number: YB/T 4002). Grades of central shrinkage cavity and porosity less than 1.0 increased significantly after application of F-PMS. This means that the inner quality of the billet was improved.

The effect of F-PMS on segregation is the same as that of F-EMS. Centerline segregation was caused by enrichment of the solute element in liquid phase at the solidification front during growth of columnar grains. Application of F-PMS drives the liquid phase of solid/liquid interface at the solidification front to flow, which then changes the temperature gradient, boundary thickness of solidification front, and mushy zone shape, and further breaks the bridge between dendritic grains, promoting grain transformation from the columnar to the equiaxed, homogenized composition and temperature of the liquid phase at the solidification front. The segregation is suppressed eventually. Additionally, F-PMS is supposed to have better control of segregation of a larger section billet compared with F-EMS due to its greater magnetic density. The energy consumption of F-PMS was also evaluated using industrial data; it is only 1/7 of F-MES, and so F-EMS has a great advantage in both segregation control and energy saving during continuous casting of high-carbon steel.

### 2.4. Control of Central Segregation Using Soft Reduction

**Figure 12:**
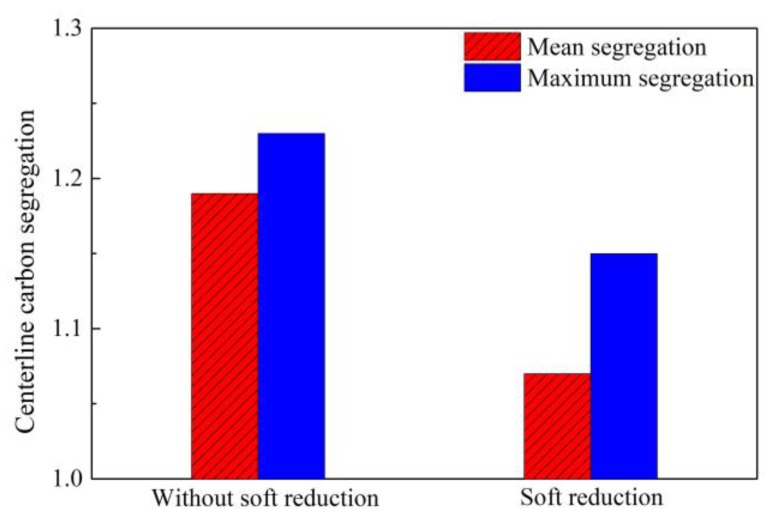
Effect of soft reduction on centerline carbon segregation of 82A steel billet. Adapted from [37].

**Figure 13:**
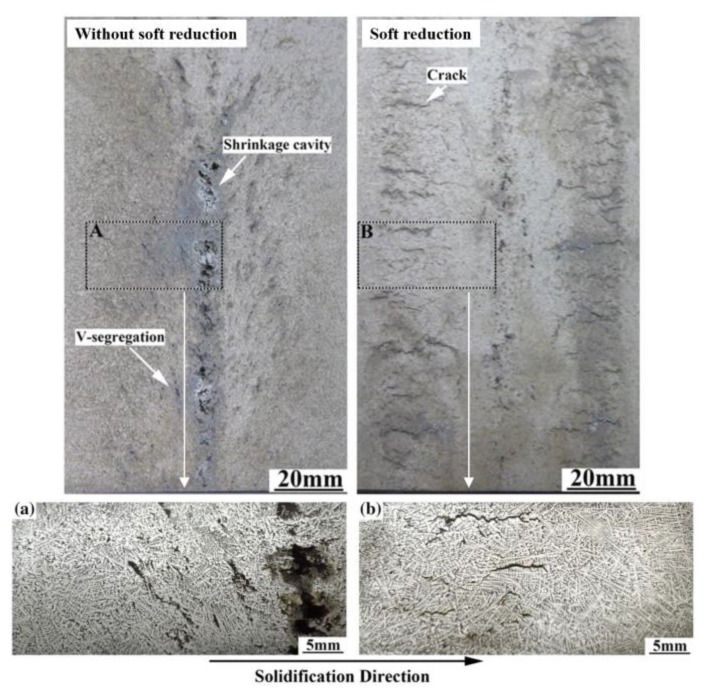
Effect of soft reduction on macro and micro structures of 82A steel billet. (**a**) Enlarged structure from A zone of billet produced without soft reduction, (**b**) Enlarged structure from B zone of billet produced with soft reduction. The pictures were taken using a digital camera after etching the steel using acid. Adapted from [37].

It is generally acknowledged that soft reduction is a good method, especially for large section bloom during two-stage hot rolling, to control centerline segregation through compensating for solidification contraction and preventing the flow of the solute-rich liquid phase into the bloom center. The soft reduction was also applied initially in our study to control segregation [51,52,53,54]. The centerline carbon segregation of 82A steel billet with a section of 180 × 240 mm is shown in Figure 12 before and after sort reduction [37]. The mean and maximum centerline carbon segregation present a great decrease from 1.19 and 1.23 to 1.07 and 1.15, respectively. As shown in Figure 13 [37], the application of soft reduction also improved the V segregation and shrinkage cavity of 82A steel billet, but some cracks also resulted, which may cause poor product quality if they cannot be eliminated during the following hot rolling.

### 2.5. Comparison in Control of Segregation

**Figure 14:**
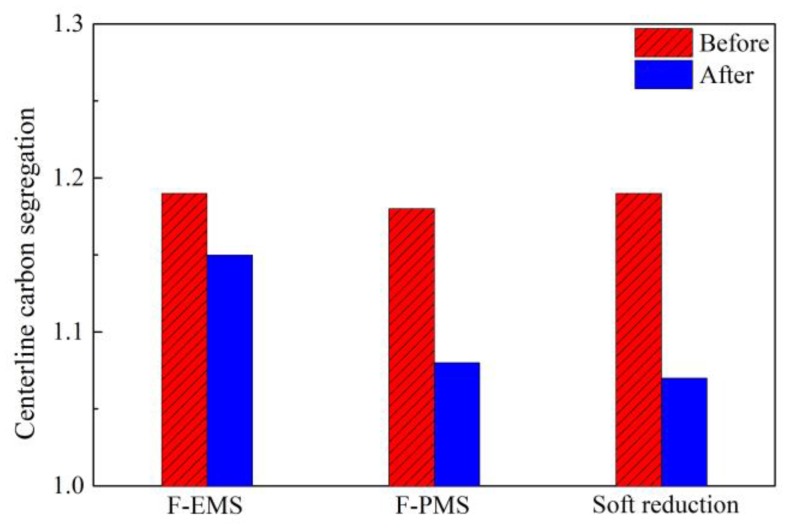
Segregation of 82A steel billet under different conditions. Adapted from [37,51].

As reviewed above, F-EMS, F-PMS, and soft reduction were applied to control segregation for quality improvement of high-carbon steel with different sections. The effectiveness was evaluated to compare their advantages in control of segregation of high-carbon steel billets with large sections. Figure 14 shows the changes of centerline carbon segregation of an 82A steel billet with a section of 180 × 240 mm under different control modes [37,50,51]. It is clear that F-PMS and soft reduction can decrease carbon segregation to a lower value. Meanwhile, F-PMS has a higher advantage in energy consumption than F-EMS. Compared with F-PMS, soft reduction shows a better effect in controlling the segregation of 82A steel with a larger section, as shown in Figure 10 and Figure 12. Despite this, soft reduction caused inner cracks, which were not found in the billet after F-PMS. 

## 3. Control of Inclusions

**Figure 15:**
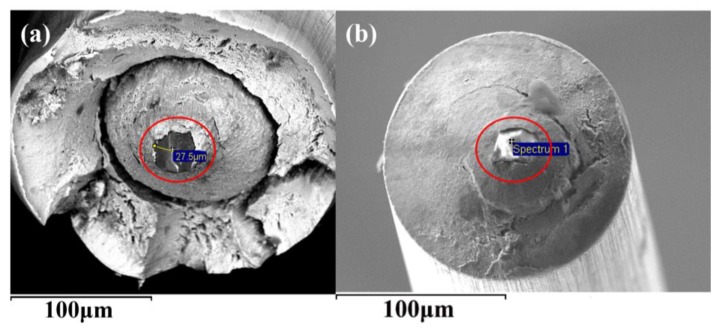
Al_2_O_3_ inclusions at center of drawing fracture obtained using SEM (**a**) steel tire cord and (**b**) saw wire. Adapted from [2,55].

Undeformable inclusions like hard high-Al_2_O_3_ inclusions, MgO·Al_2_O_3_ inclusions and some other high-melting-temperature brittle inclusions are also extremely detrimental to tire cord steel and saw wire steel drawability and service performance, because these inclusions are unlikely to elongate and likely to fracture during hot rolling and cold working [1]. As shown in Figure 15 [2,55], Al_2_O_3_ inclusions were found on the drawing fracture of steel tire cord and saw wire. Thus, eliminating these inclusions or modifying them into deformable ones is of crucial importance for the production of these high-carbon steel products. In order to improve drawing and service performance, reducing these undeformable inclusions in liquid steel, transforming them into deformable inclusions, and promoting their removal are primary goals.

### 3.1. Control of Inclusions through Redesigning SEN

**Figure 16:**
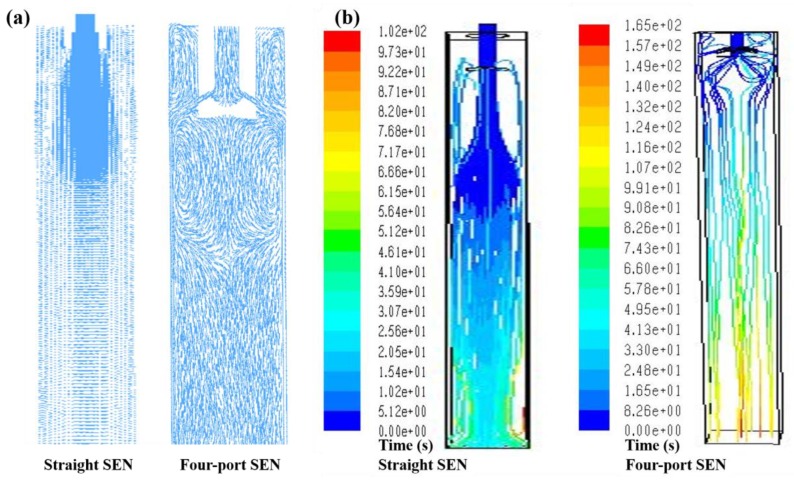
Flow field and residence time of molten steel though different submerged entry nozzles (SENs). (**a**) Flow field, (**b**) residence time. Adapted from [13].

**Figure 17:**
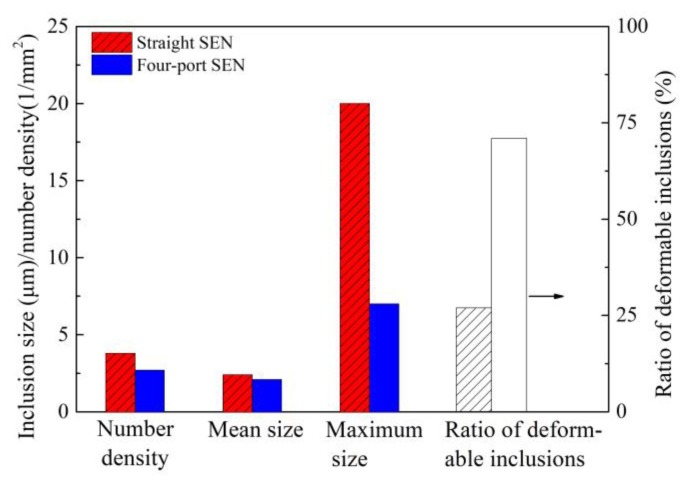
Inclusion comparison of billet cast though different SENs. Adapted from [13].

The geometry of submerged entry nozzle (SEN) can affect the fluid flow pattern of liquid steel in a continuous casting mold, which is influential in the flotation removal of inclusions. In the initial casting of high-carbon steel billet, straight SEN was always used, which would cause large impact depth and therefore go against the inclusion flotation. Figure 16 [13] illustrates a comparison of conventional straight SEN and redesigned side four-port SEN in terms of the flow field and residence time of molten steel in the mold. Compared with straight SEN, the molten steel casting through four-port SEN forms symmetrical swirling flow, while the impact depth of molten steel decreases from 330 to 260 mm and the residence time increases from 102 to 165 s; these changes are beneficial for inclusion flotation and then removal. The industrial results are also presented in Figure 17 [13]. A comparison of the number, mean size, and maximum size of inclusions obtained based on SEM images reveals that all these indexes experience a reduction when the ratio of deformable inclusions increases. It is clear that redesign of SEN accelerates inclusion removal. The ratio of deformable inclusions increases in the case of using four-port SEN, which is related to inclusion shape and flow field. (1) Large-size inclusion is easy to float according to the Stokes equation; (2) low-melting-point inclusion can gather to form large-size inclusion to float; (3) however, those small high-melting-point and undeformable inclusions float slowly, especially when using straight SEN, the residence time is very short, so those small and undeformable inclusions which cannot gather to form large ones do not have enough time to float, and these inclusions have to be left in the steel. When using four-port SEN, even small undeformable inclusions also have more time to float. However, when low-melting-point and deformable inclusions are very few, the collision probability decreases, so these small deformable inclusions are also left in the steel. 

### 3.2. Control of Inclusions through Refractory

Non-deformable inclusions should be eliminated as much as possible during the production of high-carbon steel wires. In addition to control dissolved [Al] from alloys to a low value, refractory is also an important source of these inclusions. Erosion of refractory caused by slag or steel may introduce inclusions. Zhao [15] from this group investigated the effect of different refractories on the inclusion of tire cord steel using pure MgO, ZrO_2_, SiO_2_, and Al_2_O_3_ crucibles during refining. The results indicated that the inclusions refined using a MgO crucible showed relatively better deformability than other crucibles. Therefore, MgO-based refractory was recommended for industrial application.

**Figure 18:**
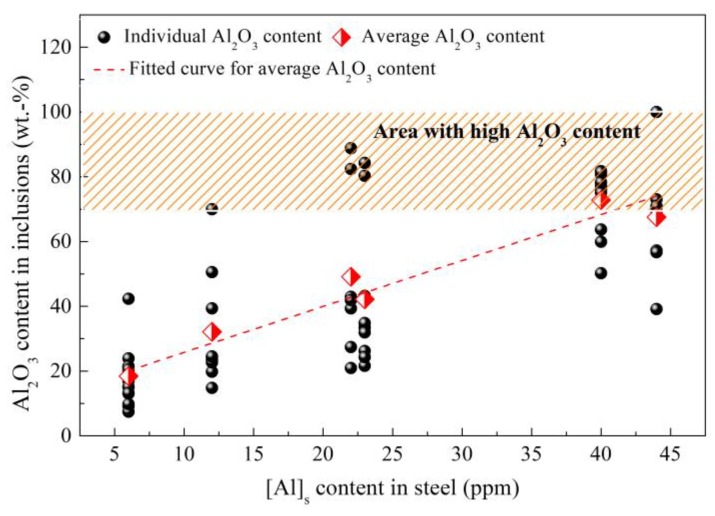
Relationship between dissolved [Al] in steel and Al_2_O_3_ content in inclusions. Adapted from [56].

**Figure 19:**
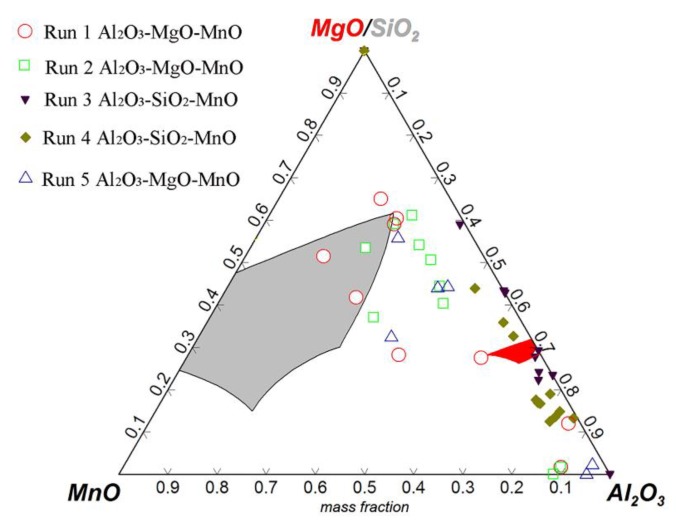
Distribution of inclusions obtained under decreasing vacuum pressure. Adapted from [56].

Despite using MgO-C refractory and controlling the Al content in raw materials and alloys to an extremely low level during industrial production, there are still some Al_2_O_3_ inclusions that can be found in saw wire steel or spring steel. The cause was checked and then the interaction of refining slag/saw wire steel/industrial MgO-C refractory under decreasing pressure was investigated due to the vacuum degassing (VD) process employed during the production of these steel grades [10,56,57]. The results show that with the decrease of vacuum pressure to 10 MPa, the dissolved [Al] in steel increases from an initial 6 to ~40 ppm [56]. As shown in Figure 18, the mean Al_2_O_3_ content in inclusion also goes up significantly from 18 to ~70 wt.%, which drives the inclusions to move into the high-melting-temperature zone of the phase diagram (Run 3 (100 MPa) and Run 4 (10 MPa) shown in Figure 19) [56]. As a result, the ratio of non-deformable inclusions increases. 

**Figure 20:**
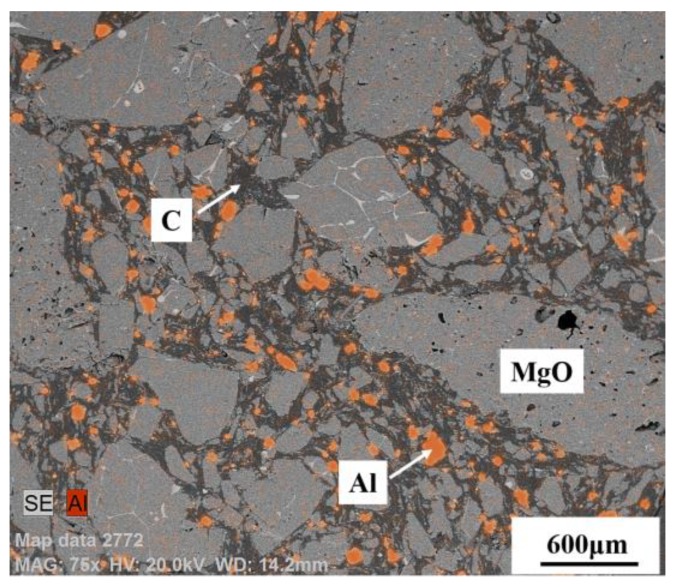
SEM image of MgO-C refractory used in the vacuum degassing (VD) process. Al and C distribute among MgO. Adapted from [56].

The cause of the increase in dissolved [Al] and subsequent generation of high Al_2_O_3_ inclusions is the Al antioxidant in the MgO-C refractory, as shown in Figure 20 [56], Al antioxidant distributed among MgO and C. The intensive reaction between MgO in refractory and [C] from steel under ever-decreasing vacuum pressure during the VD process provides a channel for Al dissolution into steel. The reaction between dissolved [Al] and [O] generates Al_2_O_3_, which further causes generation of Al_2_O_3_-rich inclusions, as shown in Figure 18. Therefore, an Al-free refractory was utilized under vacuum refining to avoid the introduction of non-deformable Al_2_O_3_-rich inclusions or refined steel without vacuum treatment.

### 3.3. Control of Inclusions through Redesigning Refining Slag

Generally, inclusions in tire cord steel or saw wire steel include MnO-SiO_2_-Al_2_O_3_ and CaO-SiO_2_-Al_2_O_3_ systems from deoxidation and refining slag, respectively. Controlling them in a reasonable zone containing ~20 wt.% Al_2_O_3_ in the corresponding phase diagrams, according to [58,59,60], to increase deformability through refining is a crucial step in decreasing their harm to final products. Consequently, reasonable design of refining slag plays an important role in this modification process of inclusions. 

**Figure 21:**
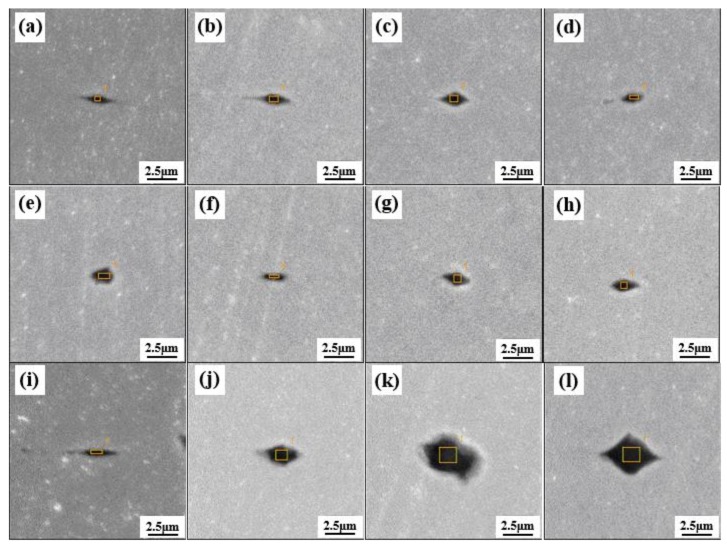
SEM images of inclusion morphology refined using different slags. (**a**–**d**) refined using slag with basicity = 1.02, 2.8 wt.% Al_2_O_3_; (**e**–**h**) refined using slag with basicity = 0.92, 6.5 wt.% Al_2_O_3_; (**i**–**l**) refined using slag with basicity = 1.08, 12 wt.% Al_2_O_3_. Adapted from [61].

**Figure 22:**
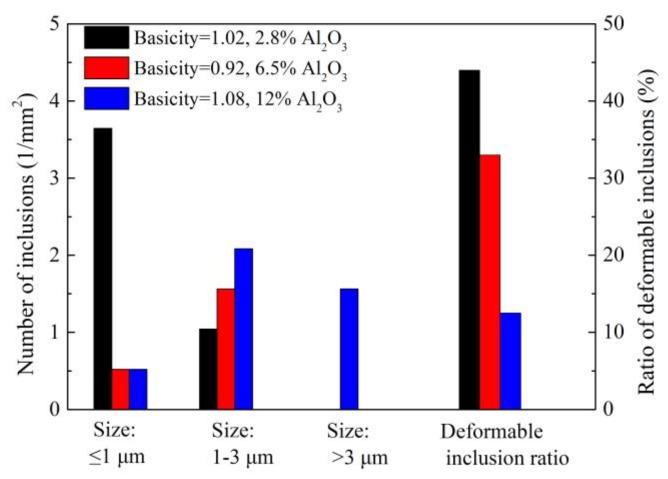
Inclusion characteristic refined using different refining slags. Adapted from [61].

**Figure 23:**
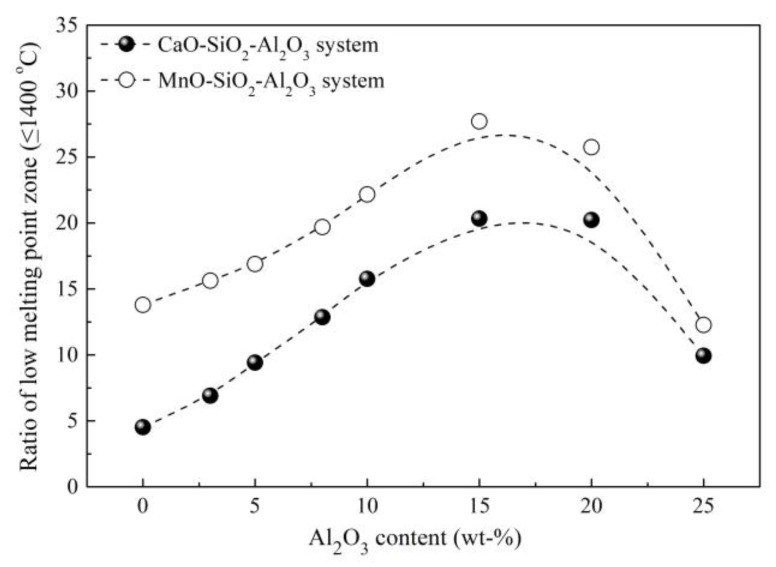
Relationship between low-melting-point zone ratio and Al_2_O_3_ contents of inclusions. Adapted from [61].

**Figure 24:**
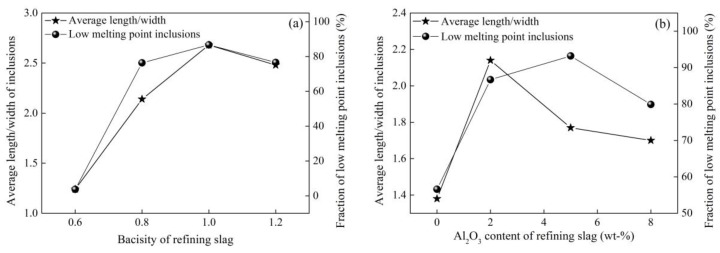
Effect of (**a**) basicity and (**b**) Al_2_O_3_ content of refining slag on inclusion deformability. Adapted from [2].

Zhao [15,61] from this group investigated the effect of Al_2_O_3_ content and basicity (defined as mass ratio of CaO to SiO_2_ in refining slag) of refining slag on the inclusion deformability of tire cord steel through industrial experiments. As shown in Figure 21 and Figure 22, the morphology of inclusions detected through SEM changes with Al_2_O_3_ content and the basicity of refining slag. The inclusions show a smaller size and have a larger ratio of deformable inclusions when the Al_2_O_3_ content and basicity of refining slag are 2.8 wt.% and 1.02 compared with the other two refining slags. As shown in Figure 23, the relationship between Al_2_O_3_ content in detected inclusions and the corresponding areas of low-melting-point zone of inclusions in the phase diagram demonstrates that inclusions have better deformability when the Al_2_O_3_ content of the inclusion is 15–20 wt.%, which is consistent with the results from [60]. Chen [2] from this group obtained similar results in a study on inclusions in saw wire steel. As shown in Figure 24, the inclusions obtained through refining slag with 2 wt.% Al_2_O_3_ and a basicity of 1.0 show better deformability, characterized by the high average length/width ratio and high ratio of low-melting-point inclusions. 

### 3.4. Control of Inclusions through Using Low-Melting-Point Compounds

Compared with tire cord steel, saw wire steel requires higher deformability for inclusions because the steel is drawn into a wire with a diameter as low as 0.08 mm. Conventional refining using synthetic slag has a limited ability to modify inclusions in saw wire steel to the demanded level. Therefore, some compounds like B_2_O_3_ and alkali metal compounds were used to modify inclusions in consideration of their low melting points.

**Figure 25:**
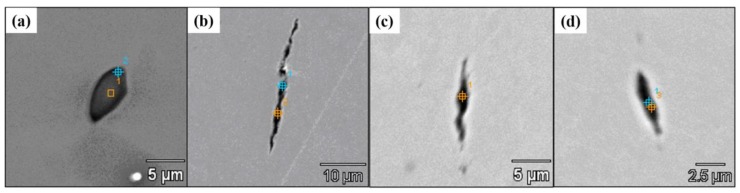
SEM images of inclusions (**a**) ellipsoidal inclusion before addition of B_2_O_3_, and (**b**–**d**) strip-like inclusions after addition of B_2_O_3_. Adapted from [62,63].

**Figure 26:**
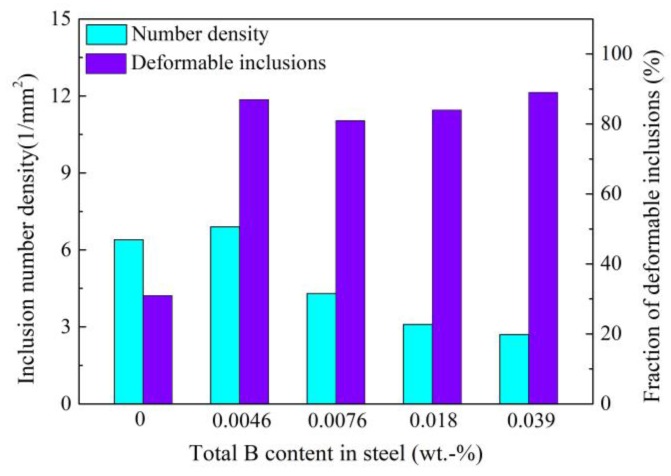
Effect of total B content on inclusion number density and deformability. Adapted from [62,63].

Cui [62,63] of this group investigated the effect of addition of B_2_O_3_ into refining slag on inclusions in tire cord steel. As shown in Figure 25, compared with inclusions obtained before B_2_O_3_ addition (Figure 25a), the inclusions achieve great deformation after B_2_O_3_ addition (Figure 25b–d). It is difficult to determine the B content in inclusions using SEM-EDS analysis, so the total B content and acid-soluble B content in steel were measured and we found that about 30 wt.% B was incorporated into inclusions in the form of B_2_O_3_. This indicates that the introduction of B_2_O_3_ into inclusions improves their deformability. Furthermore, as illustrated in Figure 26, the addition of B_2_O_3_ also decreases inclusion number density and improves the fraction of deformable inclusions significantly.

**Figure 27:**
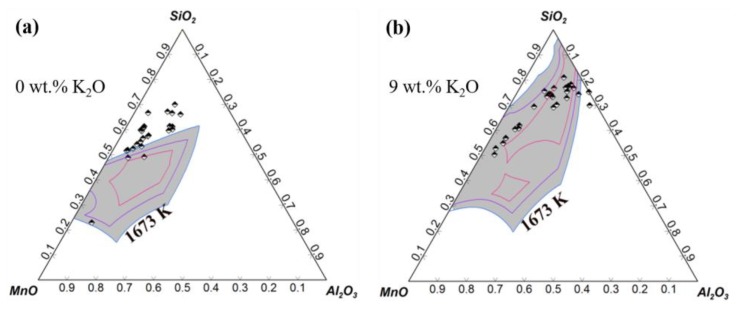
Effect of K_2_O content on inclusion distribution in phase diagram. (**a**) 0 wt.%, (**b**) 9 wt.%. Adapted from [64].

**Figure 28:**
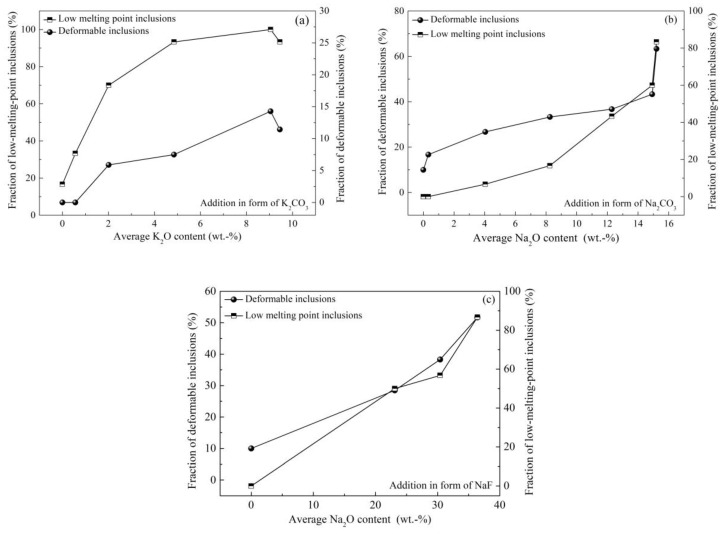
Effect of K_2_O and Na_2_O contents of inclusions on inclusion deformability. (**a**) Addition in the form of K_2_CO_3_, (**b**) addition in the form of Na_2_CO_3_, (**c**) addition in the form of NaF. Adapted from [2,64,65].

The addition of low-melting-point alkali metal compounds into inclusions to modify them was attempted by some researchers, but this was failed because the addition of alkali metal into molten steel causes serious splashing and evaporation. Chen of this group achieved the successful addition of alkali into steel and then inclusions, and investigated its effect on inclusions in saw wire steel and spring steel [2,64,65,66]. As shown in Figure 27 [64], the addition of K_2_CO_3_ in refining slag generates inclusions containing K_2_O; almost all inclusions move into the low-melting-point zone in the phase diagram with an increase of up to 9 wt.% in the average K_2_O content of inclusions. Figure 28 [2,64,65] compares the effects of alkali oxides in inclusions on inclusion characteristics; these inclusions were modified by mixed additions of iron powder and alkali metal in the form of K_2_CO_3_, Na_2_CO_3_, or NaF, respectively. It is clear that fractions of low-melting-point inclusions and deformable inclusions increase with an increase in the K_2_O and Na_2_O contents in inclusions. Compared with inclusions modified by K_2_CO_3_, the inclusions modified by Na_2_CO_3_ show better deformability. However, the addition of carbonates causes splashing to a certain extent; the addition of NaF can inhibit this phenomenon and therefore increase the yield of Na_2_O in inclusions, which further improves the deformability.

In this section, inclusions obtained after various treatments are evaluated and some measurements to reduce inclusion amount and improve the deformability are discussed, and prove to be effective. During this process, the inclusions were evaluated through two-dimensional analysis using SEM images. The deformability was evaluated according to an international standard ISO 4967 and a China standard GB/T-10561, which recommend a value of length/width ratio of three to define the deformability. One point should be noted with respect to the evaluation method, that a three-dimensional analysis should be more accurate than a two-dimensional analysis because the two-dimensional analysis may not reflect the section of inclusion with largest area. Therefore, an electrolytic separation method was recommended to reflect the three-dimensional morphology of inclusions. The criteria for inclusion deformability using a length/width of three is not completely accurate because we found several brittle inclusions also present a high length/width ratio. However, in addition to this method, there is currently no better and more uniform method to evaluate deformability of inclusion.

## 4. Control of Microstructure

### 4.1. Control of Pearlite, Network Cementite, and Martensite

**Figure 29:**
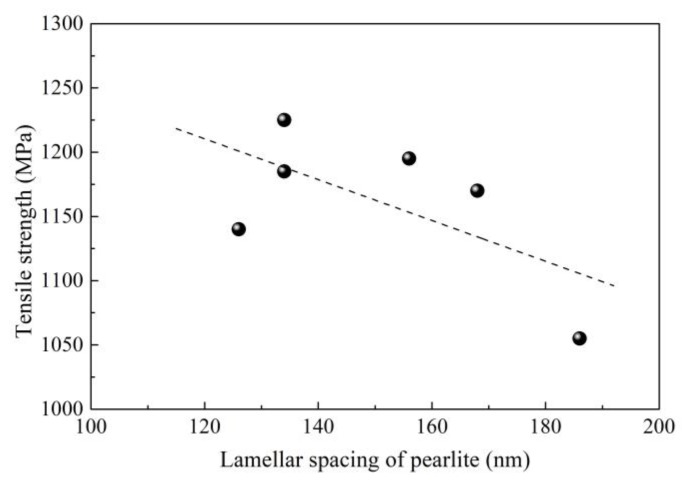
Effect of lamellar spacing of pearlite on tensile strength of 82B steel wire rod. Adapted from [38].

**Figure 30:**
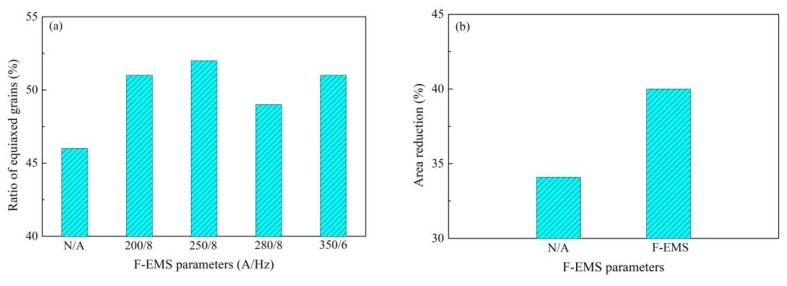
Effect of F-EMS on (**a**) equiaxed grains and (**b**) area reduction of 82B steel. The equiaxed grain structure was obtained based on optical microscope images, area reduction was obtained based on dynamic thermal-mechanical testing of materials and simulation of processes using the Gleeble testing system. Adapted from [29].

High-carbon steel wires like steel stranded wire, steel tire cord and saw wire require high strength due to their application fields. For example, a tensile strength as high as 4000 MPa is required by saw wire [1]. As described above, undeformable inclusions are detrimental to the strength; many measurements have been implemented to eliminate or modify them during our and previous investigations. Additionally, the structure is also a key feature that should be controlled to further improve the strength and ductility. Fully fine pearlite microstructure, as a laminated composite of ferrite and cementite, is a desirable target of high-carbon steel wire because of its excellent combination of strength and ductility [67]. The higher fraction and smaller lamellar spacing of the pearlite microstructure indicates higher strength, which can be achieved through the refinement of grains and reduction of transformation temperature from austenite. As shown in Figure 29 [38], the reduction of lamellar spacing of pearlite microstructure enhances the tensile strength of 82B steel wire. Application of F-EMS can refine grains, increase the ratios of equiaxed grains and area reduction (Figure 30 [29]), which means an increase in strength and ductility. 

**Figure 31:**
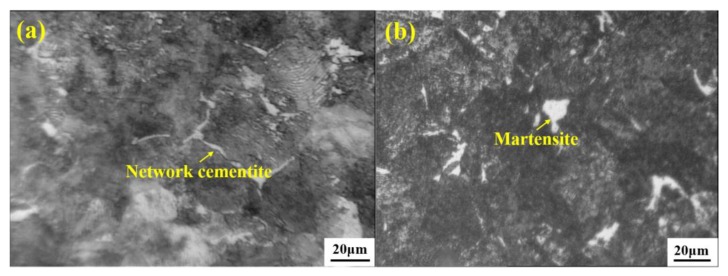
(**a**) Network cementite distributes along the grain boundary, and (**b**) island-like martensite in a wire rod of tire cord steel obtained using an optical microscope (OM). Adapted from [68].

However, at the same time, despite cementite being necessary for the formation of the desired fine pearlite microstructure, the network cementite formed and distributed along the grain boundary (Figure 31a [68]) should be eliminated as much as possible because these are preferred sites for fracture initiation and propagation. Ultimately, the formation of network cementite can be attributed to the centerline carbon segregation of the billet and the slow cooling rate from A_cm_ to A_1_ temperature [18]. As reviewed above, many measurements like intensive secondary cooling, F-EMS, F-PMS and soft reduction have been investigated and applied to control centerline carbon segregation. In order to increase the cooling rate, forced-air cooling or salt-bath cooling was utilized. Eventually, the network cementite can be controlled to a low level through a combination of the above measurements.

In addition to network cementite, the martensite as in Figure 31b is also a potential origin of cracking during drawing due to its high strength and hardness [18]. He [69] from this group measured the nanohardness of martensite at 8.9 GPa by nanoindentation, which is far larger than that of fine pearlite, 4.5 GPa. Precipitation of martensite is close to the cooling rate. A critical cooling rate was found at which martensite transformation starts. Apparently, controlling the cooling rate to be less than the critical cooling rate can suppress the precipitation of martensite. The critical cooling rate depends on steel compositions, which can be determined through constructing continuous cooling transformation curves. Cui [63] of this group reported that the critical cooling for 72A tire cord steel is ~1000 °C/min. Precipitation of martensite is also related to the segregation of Mn and Cr. He [69] compared the contents of Mn and Cr of martensite with the surrounding pearlite microstructure of 82B steel, and found that martensite has almost double the Mn and Cr contents (1.74 wt.% Mn-0.35 wt.% Cr), relative to the levels in the pearlite microstructure (0.79 wt.% Mn-0 Cr), which indicates intensive segregation of Mn and Cr in martensite. A positive correlation between Mn, Cr, and C was found, and it was also found that F-EMS can remarkably hamper the segregation of Mn and Cr, and the subsequent precipitation of martensite.

### 4.2. Control of Banded Structure

**Figure 32:**
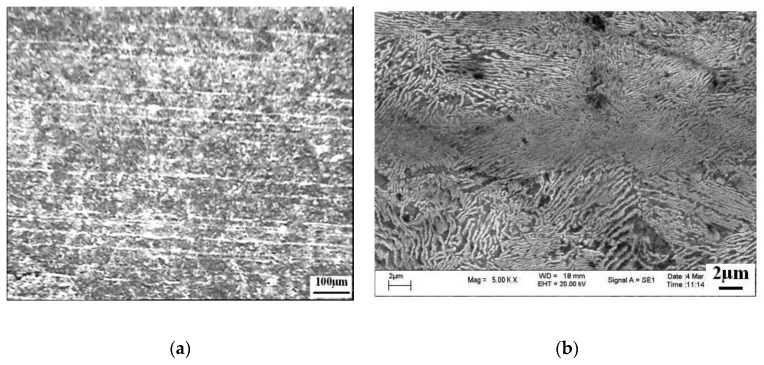
SEM images of the banded structure in a wire rod of tire cord steel. (**a**) Appeared macroscopically in the form of fiber or band, (**b**) appeared microscopically in pearlite with small lamellar spacing. Adapted from [23].

A banded structure, as shown in Figure 32 [23], is a kind of pearlite microstructure that always gathers in the center of high-carbon steel wire rods; it appears in white fiber macroscopically but in pearlite with especially small lamellar spacing microscopically. Cui [23] from this group found that a banded structure has a higher P content (~0.125 wt.% P) than the surrounding microstructure (~0 wt.% P) detected using an electron probe micro-analyzer (EPMA), and a higher nanohardness of 5.7 GPa than that of the surrounding microstructure (4.8 GPa). This hard structure is the origin of fracture initiation and propagation during drawing. Further study revealed P segregates among secondary dendrites, especially at the interface of the columnar grain zone and the equiaxed grain zone. Promotion of P diffusion using a higher heating temperature was investigated and we found that the banded structure was inhibited to some extent but not completely eliminated. It can therefore be concluded that the banded structure was mainly caused by P segregation that is difficult to diffuse during hot rolling. 

**Figure 33:**
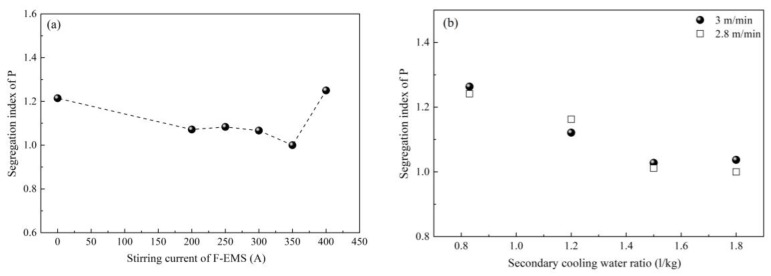
Effect of (**a**) F-EMS and (**b**) secondary cooling and casting speed on P segregation. Adapted from [63].

**Figure 34:**
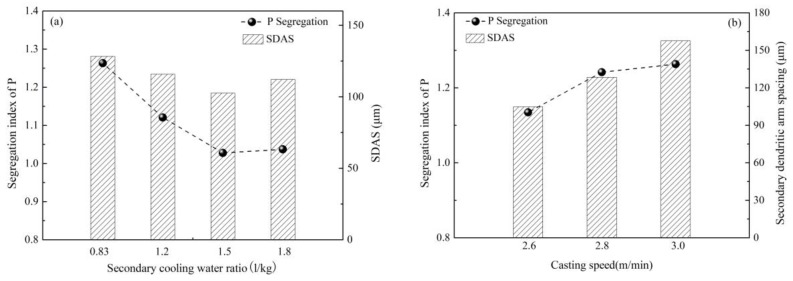
Effect of (**a**) secondary cooling and (**b**) casting speed on SDAS and P segregation. Adapted from [63].

Based on the above, it is crucial to control P segregation during casting. The increase of secondary cooling water ratio and application of F-EMS, as in Figure 33, were investigated and found to inhibit segregation of P [63,70]. Further study, as shown in Figure 34, found that segregation of P has a positive correlation with SDAS [63]. An increase of casting speed promotes P segregation, accompanied by an increase of SDAS, but the increase in secondary cooling water ratio inhibits the segregation of P, accompanied by a decrease of SDAS. The above study indirectly verifies that P segregates among secondary dendrites; those measurements that can decrease SDAS can also inhibit P segregation and thus the banded structure.

### 4.3. Control of Texture

**Figure 35:**
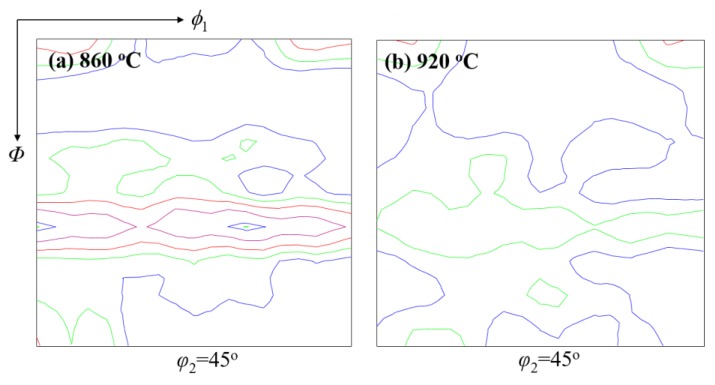
Texture of steel tire cord from different laying temperatures. Texture was obtained by Electron Back-Scattered Diffraction (EBSD). Adapted from [24].

**Figure 36:**
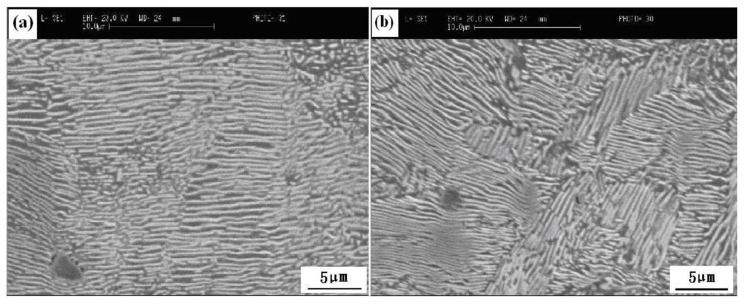
SEM images of pearlite lamellar spacing of steel tire cord from different laying temperatures (**a**) 860 °C, distributed orderly, (**b**) 920 °C, distributed randomly. Adapted from [24].

Mechanical properties like the grain orientation of steel wire during cold drawing are also correlated with the microstructure. High-carbon steel wire has a body-centered cubic structure under cold drawing temperature, which is characterized by the glide plane {110} texture with the highest atom close-packed density [71]. The other textures like {112}, {123}, and especially {111} of the axial plane, are difficult to slip during drawing due to low atom density. The desired stress used to make grains slip during cold drawing will rise greatly if the fraction of {111} texture of axial plane increases, which will cause strong drawing resistance to stand up to further drawing of steel wire. A breakage will result if the fraction of {111} of axial plane reaches a critical value at which the drawing stress is larger than the tensile strength of the steel wire. Cui [24] from this group investigated the effect of heating temperature, heating time, and laying temperature on the texture of tire cord steel wire rod. He found a maximum intensity of {111} texture reduces from 5.3 to 4.0, but the tensile strength and area reduction obtain improvement through prolonging heating time. A decrease of maximum intensity of {111} texture from 6.3 to 3.4 along with a strength increase of 43 MPa can also be caused by increasing the heating temperature. As shown in Figure 35 and Figure 36, increasing the laying temperature from 820 to 920 °C decreases the maximum intensity of {111} texture to 3.0; meanwhile, the distribution of pearlite in a pearlitic colony becomes random, and the lamellar spacing becomes small [24,63]. A low maximum intensity of {111} texture and a random pearlite with smaller lamellar spacing indicate an improvement in the mechanical property. 

## 5. Summary

This review summarizes the control measurements of key quality issues like centerline carbon segregate, inclusions and microstructure for the production of high-carbon steel for steel strands, steel tire cords, and saw wires, which have been investigated and developed by Prof. Weiqing Chen’s group at USTB based on the low-cost “rolling in one heat” process since 2000. This series of control measurements achieved successful industrial application in more than 10 steel plants, and has also been extended to applications in spring steel, welding wire steel and some other wire rods. The major findings of this study and industrial process can be summarized as follows:

1) Intensive secondary cooling, F-EMS, F-PMS and soft reduction in control of segregation have been investigated and applied, and their application scopes have been defined. In particular, F-PMS has been developed and applied innovatively in the industrial production of high-carbon steel and shows advantages in terms of reducing the cost and improving the quality of products.
2) A combination of the redesign of SEN, the redesign of refining slag, the application of Al-free refractory during the vacuum process, and the improvement of the deformability of inclusions through the addition of B_2_O_3_ and alkali metal compounds has been investigated and applied in the industrial production of high-carbon steel, promoting the inclusion removal and greatly improving the deformability of inclusions.
3) Final stirring and using an appropriate cooling rate are beneficial for effective control of network cementite and martensite. Formation of a hard banded structure is close to P segregation, which can be controlled through effective secondary cooling, casting speed, and final stirring. Undesired {111} texture can be controlled through improvements of heating time, heating temperature, and especially, the laying temperature.
